# Arboviral Etiologies of Acute Febrile Illnesses in Western South America, 2000–2007

**DOI:** 10.1371/journal.pntd.0000787

**Published:** 2010-08-10

**Authors:** Brett M. Forshey, Carolina Guevara, V. Alberto Laguna-Torres, Manuel Cespedes, Jorge Vargas, Alberto Gianella, Efrain Vallejo, César Madrid, Nicolas Aguayo, Eduardo Gotuzzo, Victor Suarez, Ana Maria Morales, Luis Beingolea, Nora Reyes, Juan Perez, Monica Negrete, Claudio Rocha, Amy C. Morrison, Kevin L. Russell, Patrick J. Blair, James G. Olson, Tadeusz J. Kochel, for the NMRCD Febrile Surveillance Working Group

**Affiliations:** 1 United States Naval Medical Research Center Detachment, Iquitos and Lima, Peru; 2 Instituto Nacional de Salud, Lima, Peru; 3 CENETROP, Santa Cruz, Bolivia; 4 SEDES, Cochabamba, Bolivia; 5 Hospital Naval, Guayaquil, Ecuador; 6 Asociación Rayos del Sol, Asunción, Paraguay; 7 Instituto de Medicina Tropical “Alexander von Humboldt”, Universidad Peruana Cayetano Heredia, Lima, Peru; 8 Dirección General de Epidemiología, Ministerio de Salud, Lima, Peru; 9 Universidad Nacional Mayor de San Marcos, Lima, Peru; 10 University of California Davis, Davis, California, United States of America; Pediatric Dengue Vaccine Initiative, United States of America

## Abstract

**Background:**

Arthropod-borne viruses (arboviruses) are among the most common agents of human febrile illness worldwide and the most important emerging pathogens, causing multiple notable epidemics of human disease over recent decades. Despite the public health relevance, little is know about the geographic distribution, relative impact, and risk factors for arbovirus infection in many regions of the world. Our objectives were to describe the arboviruses associated with acute undifferentiated febrile illness in participating clinics in four countries in South America and to provide detailed epidemiological analysis of arbovirus infection in Iquitos, Peru, where more extensive monitoring was conducted.

**Methodology/Findings:**

A clinic-based syndromic surveillance system was implemented in 13 locations in Ecuador, Peru, Bolivia, and Paraguay. Serum samples and demographic information were collected from febrile participants reporting to local health clinics or hospitals. Acute-phase sera were tested for viral infection by immunofluorescence assay or RT-PCR, while acute- and convalescent-phase sera were tested for pathogen-specific IgM by ELISA. Between May 2000 and December 2007, 20,880 participants were included in the study, with evidence for recent arbovirus infection detected for 6,793 (32.5%). Dengue viruses (*Flavivirus*) were the most common arbovirus infections, totaling 26.0% of febrile episodes, with DENV-3 as the most common serotype. *Alphavirus* (Venezuelan equine encephalitis virus [VEEV] and Mayaro virus [MAYV]) and *Orthobunyavirus* (Oropouche virus [OROV], Group C viruses, and Guaroa virus) infections were both observed in approximately 3% of febrile episodes. In Iquitos, risk factors for VEEV and MAYV infection included being male and reporting to a rural (vs urban) clinic. In contrast, OROV infection was similar between sexes and type of clinic.

**Conclusions/Significance:**

Our data provide a better understanding of the geographic range of arboviruses in South America and highlight the diversity of pathogens in circulation. These arboviruses are currently significant causes of human illness in endemic regions but also have potential for further expansion. Our data provide a basis for analyzing changes in their ecology and epidemiology.

## Author Summary

Over recent decades, the variety and quantity of diseases caused by viruses transmitted to humans by mosquitoes and other arthropods (also known as arboviruses) have increased around the world. One difficulty in studying these diseases is the fact that the symptoms are often non-descript, with patients reporting such symptoms as low-grade fever and headache. Our goal in this study was to use laboratory tests to determine the causes of such non-descript illnesses in sites in four countries in South America, focusing on arboviruses. We established a surveillance network in 13 locations in Ecuador, Peru, Bolivia, and Paraguay, where patient samples were collected and then sent to a central laboratory for testing. Between May 2000 and December 2007, blood serum samples were collected from more than 20,000 participants with fever, and recent arbovirus infection was detected for nearly one third of them. The most common viruses were dengue viruses (genera *Flavivirus*). We also detected infection by viruses from other genera, including *Alphavirus* and *Orthobunyavirus*. This data is important for understanding how such viruses might emerge as significant human pathogens.

## Introduction

Over the past few decades there has been a global resurgence of arthropod-borne viral pathogens (arboviruses) worldwide [1], [2], particularly those transmitted by mosquitoes. Despite the public health relevance, the geographic range, relative impact, and epidemiologic characteristics associated with arbovirus infection are poorly described in many regions of the world. Arboviruses are a heterogeneous group, but those of medical relevance largely belong to a few virus genera, including *Flavivirus*, *Alphavirus*, and *Orthobunyavirus*. Prominent examples of emergent arboviruses include West Nile virus (WNV; *Flavivirus*) in North America, Japanese encephalitis virus (JEV; *Flavivirus*) in Asia, chikungunya virus (CHIKV; *Alphavirus*) in the Indian Ocean region and dengue viruses (DENV; *Flavivirus*) worldwide. One common feature shared by many emergent arboviruses is the capacity to expand host and geographical range, owing in part to the plasticity of the RNA genome [3]. Some arboviruses have evolved to exploit humans as their primary reservoir, while others rely on birds or peridomestic animals, with human infection resulting from spill-over from zoonotic replication cycles. Human exposure to sylvatic arbovirus cycles is likely to increase as activities continue to encroach on forested areas worldwide. Tropical areas in particular, with year-round hot and humid conditions, are well-suited for maintaining arboviruses with potential to emerge as significant human pathogens [4]. In the neotropics alone, greater than 145 distinct arbovirus species have been identified [4], many of which have already been associated with human illness.

One limitation of conducting surveillance for arboviral diseases is the generic nature of disease presentation. While severe disease can result, such as hemorrhagic manifestations (DENV and yellow fever virus [YFV]) or neurological disease (WNV, JEV, and Venezuelan equine encephalitis virus [VEEV]), arbovirus infection typically results in mild to moderate febrile illness [2], [5], [6]. Particularly early in disease progression, patients commonly present with undifferentiated febrile illness [5], [7] rendering clinical diagnosis unreliable [8]. In DENV-endemic areas, for example, diseases caused by co-circulating pathogens have been found to be often misclassified [8], [9]. In light of the lack of distinct clinical presentation and the diversity of the etiologic agents, laboratory support has become a critical component of effective surveillance programs.

The impact on human health in endemic regions and the potential for broader spread underscore the importance of improving understanding of arbovirus transmission patterns. Currently the epidemiological characteristics and geographic range for many endemic arboviruses in South America are poorly understood. To begin to address this gap, we established a laboratory-supported clinic-based study to identify the etiologic agents associated with undifferentiated febrile illness in sites in Peru, Ecuador, Bolivia, and Paraguay. Herein we describe the geographic distribution of distinct arboviruses and their relative contribution to human febrile illness in these study sites. In addition, we present the temporal trends and epidemiological characteristics associated with arbovirus infection in Iquitos, Peru, a site where more extensive monitoring was conducted.

## Methods

### Study Locations

**Figure 1:**
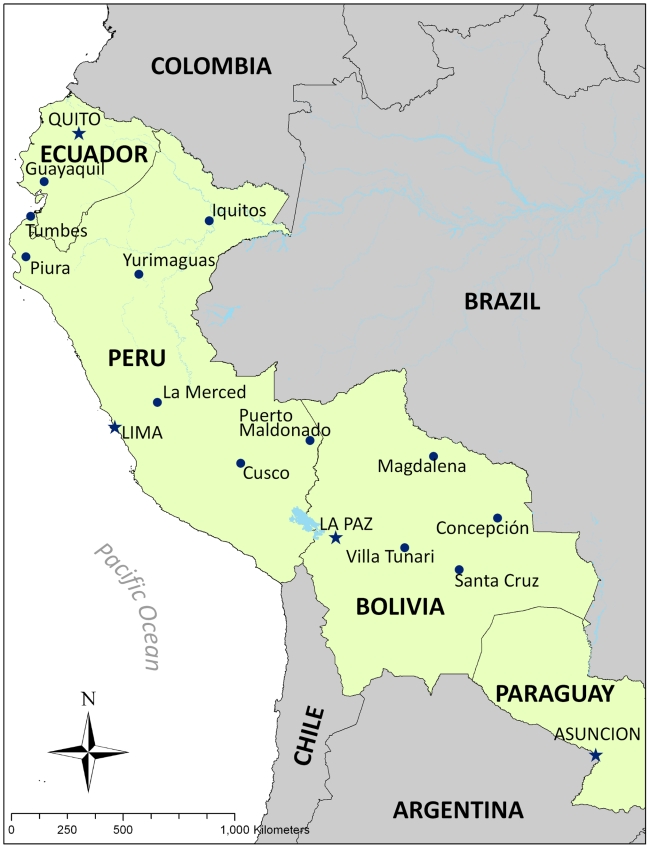
Map of study sites in Ecuador, Peru, Bolivia, and Paraguay. Capitals of Ecuador (Quito), Peru (Lima), and Bolivia (La Paz) are shown for reference.

In 1990 the U.S. Naval Medical Research Center Detachment (NMRCD) initiated a clinic-based surveillance program to determine the etiologies of febrile illness in Iquitos, Peru [10]–[12]. In 2000 NMRCD collaborated with local Ministries of Health to expand the surveillance program into other regions of Peru and South America, including sites in Ecuador, Bolivia, and Paraguay. In addition to Iquitos, in 2000 the study was implemented at regional sites in or near Piura, Cusco, Tumbes, and Yurimaguas, Peru, as well as Santa Cruz, Bolivia (Figure 1; Table 1). Additional sites were later added in Concepción, Magdalena, and Cochabamba (Villa Tunari and Eterazama), Bolivia; Guayaquil, Ecuador; Asunción, Paraguay; and La Merced and Puerto Maldonado, Perú. Participants were recruited when reporting with acute febrile illness to public, private, or military health facilities in and around these regional centers. Details of the study sites are described in Table 1. Study sites were selected based largely on locations in hot and humid climates conducive for arbovirus transmission, typically situated near or in tropical rainforest regions. Notable exceptions include Piura, Tumbes, and Cusco, which are located in coastal desert (Piura and Tumbes) or highlands (Cusco; Figure 1 and Table 1) regions. It should be noted that the study staff in Cusco (Hospital Regional) on occasion attended to participants arriving from surrounding highlands rainforest regions.

**Table 1:** Febrile illness surveillance study sites.

Location	Year^1^	No. and types of centers^2^		Alt (m)	Mean annual temp (°C)	Mean annual precip. (cm)
**Bolivia**						
Concepción	2004	1	H	497	24.4	116.3
Magdalena	2004	1	H	193	26.7	140.3
Santa Cruz	2000	2	H	437	24.5	134.6
Cochabamba	2005	2	H, C	265	24.6	585.0
**Ecuador**						
Guayaquil	2003	1	M	4	25.0	108.0
**Paraguay**						
Asunción	2006	1	P	89	23.2	136.6
**Peru**						
Cusco	2000	1	H	3248	12.3	67.1
Iquitos	2000	13	M, C, H	125	26.1	287.9
La Merced	2001	1	H	751	23.0	237.9
Piura	2000	2	C	49	24.5	6.7
P. Maldonado	2004	3	H, C	265	25.3	226.0
Tumbes	2000	2	C	25	25.2	15.0
Yurimaguas	2000	1	H	179	26.6	207.1

1 Year site was added to the study.

2 Military (M), Public Hospital (H), Public Health Clinic (C), Private Hospital (P).

### Ethics statement

Study protocols (NMRCD.2000.0006 [Peru], NMRCD.2001.0002 [Ecuador], NMRCD.2000.0008 [Bolivia], and NMRCD.2005.0008 [Paraguay]) were approved by the Naval Medical Research Center Institutional Review Board (Bethesda, MD) in compliance with all U.S. Federal regulations governing the protection of human subjects. In addition, the study protocols were reviewed and approved by health authorities in Peru (Dirección General de Epidemiología), Bolivia (Servicio Departamental de Salud, Santa Cruz and Colegio Medico de Santa Cruz), Ecuador (Ministerio de Salud Publica, Comando Conjunto de la Fuerzas Armadas, and Escuela de Sanidad in Guayaquil) and Paraguay (Ministerio de Salud y Bienestar Social and Comité de Ética de Asociación de Rayos de Sol). Written consent was obtained from patients 18 years of age and older. For patients younger than 18 years, written consent was obtained from a parent or legal guardian. Additionally, written assent was obtained from patients between 8 and 17 years of age.

### Study subjects

Study subjects included patients 5 years of age or older who presented in outpatient clinics or hospitals with acute, undifferentiated, febrile illness (greater than or equal to 38°C for 7 days duration or less) along with one or more of the following symptoms: headache, muscle, ocular and/or joint pain, generalized fatigue, cough, nausea, vomiting, sore throat, rhinorrhea, difficulty breathing, diarrhea, jaundice, dizziness, disorientation, stiff neck, or bleeding manifestations. Children younger than five years of age were included if they presented with hemorrhagic manifestations indicative of dengue hemorrhagic fever (DHF), including epistaxis, pleural effusion, platelets less than 100,000/ml, petechiae, or bloody stool or vomit. Exclusion criteria included fever in excess of seven days or an identifiable focus of infection, such as sinusitis, pneumonia, acute otitis media, or acute urinary tract infection. Demographic data, medical history, and clinical features for each patient were obtained using a standard questionnaire. In malaria-endemic regions if malaria was suspected, capillary blood from febrile patients was screened for *Plasmodium* spp. by clinic or hospital personnel according to routine diagnostic procedures at each site. Peripheral blood samples were screened by microscopic analysis of stained thick smear slides. In some sites, owing to the possibility of arbovirus co-infection, malaria-positive patients were subsequently invited to participate in the NMRCD study, with malaria results recorded along with symptoms and demographic information.

During the acute phase of illness blood samples were obtained from each patient, and when possible, convalescent samples were obtained 10 days to 4 weeks later for serological studies. For patients older than 10 years of age, up to 15 mL of blood was collected, and for patients younger than 10 years of age, up to 7 mL of blood was collected. Trained phlebotomists collected blood samples via arm venipuncture using standard methods and universal precautions.

### Laboratory Procedures

#### Virus isolation

Acute-phase serum samples were transported on dry ice to the U.S. NMRCD laboratory in Lima and stored at −80°C. Serum samples were thawed and diluted 1∶10 in Eagle's minimum essential medium (EMEM) containing 2% heat-inactivated fetal bovine serum and antibiotics (200 U/ml penicillin and 200 µg streptomycin). African Green Monkey Vero (37°C) and *Aedes albopictus* C6/36 (28°C) cell cultures were each inoculated with 200 µl of the diluted serum in 25 ml flasks. Upon observation of cytopathic effect (CPE) with a light microscope, or ten days post-inoculation if no CPE was observed, cells were removed from the flasks, collected by centrifugation for 10 minutes, and placed on 12-well glass spot-slides for microscopic examination by standard indirect immunofluoresence assay (IFA). Viral antigens were detected with hyperimmune mouse ascitic fluid (HMAF) produced in the NMRCD-Lima laboratory against the arbovirus isolates listed below, followed by the addition of fluorescein-conjugated goat anti-mouse IgG, similar to previously described [11], [13], [14]. DENV serotypes were identified using serotype-specific monoclonal antibodies (DENV-1: 15F3, DENV-2: 3H5; DENV-3: 5D4; DENV-4: 1H10).

#### Serology

IgM titers were determined by using IgM-capture enzyme-linked immunosorbant assays (ELISA), as previously described [15], [16]. Briefly, microtiter plates (96-well format) were coated with anti-human IgM (Tago, Inc., Burlingame, CA) diluted 1∶800 in phosphate-buffered saline (PBS) and incubated overnight at 4°C. Participant serum was diluted 1∶100 and incubated in coated wells for 1 h at 37°C, followed by the addition of viral antigen and incubation at 37°C for 1 h. Viral antigens were detected with HMAF (produced by inoculation of mice with the respective viral strains), followed by horseradish peroxidase-conjugated goat anti-mouse IgM + IgG (Pierce, Rockford, IL). Following the addition of ABTS (2,2′-azino-bis-[3-ethylbenzthiazoline-6-sulfonic acid]) colorimetric substrate, plates were read at 410 nm with a Dynex ELISA MRX Revelation absorbance reader (Dynex Technologies, Chantilly, VA). All acute- and convalescent-phase samples were initially screened at 1∶100. Previously identified reactive patient sera were used as positive controls, and normal human serum was used as a negative control. Samples exceeding the reference cut-off value, calculated as the mean of seven antibody-negative samples (normal human serum) plus three standard deviations, were considered IgM-positive. Positive samples were subsequently re-tested at four-fold serial dilutions (1∶100, 1∶400, 1∶1600, and 1∶6400). The highest dilution at which the OD still exceeded the cut-off value was considered the final titer.

Viral antigens for the ELISAs were produced at the NMRCD laboratory in Lima. Antigens were derived from suckling mouse brain or from supernatants collected from infected African green monkey kidney Vero cells inoculated with the following virus strains: for VEEV, subtype ID strain IQT8131; for Murutucu Virus (MURV), strain IQT9891; for Caraparu Virus (CARV), strain IQU1719; for MAYV, genotype D strain TRVL15537; for Eastern equine encephalitis virus (EEEV), strain VR65, for YFV, 17D strain OBS5926; for Oropouche virus (OROV), strain 172; for Guaroa virus (GROV) strain FSJ1340; for DENV, antigen derived from pooled supernatants from infected Vero cells cultures of all four serotypes, including DENV-1 West Pac 74, DENV-2 S16803, DENV-3 CH53489, and DENV-4 TVP-360. Prior to homogenization, antigen preparations were inactivated using 3 mM binary ethylenimine.

#### RT-PCR

To confirm results from IFA, a subset of acute-phase serum samples were tested for the presence of pathogen-specific nucleotide sequences by reverse transcription polymerase chain reaction (RT-PCR). DENV sequences were first amplified using pan-DENV primers followed by serotype-specific nested primers, as described previously [17]. VEEV sequences were amplified as previously reported in [18] and [12], with primer pair V8369(+) and V9207B(−) or V9257(−) used to detect VEEV RNA. MAYV sequences were amplified with primer pairs 7336(+)/8140(−) or 9368(+)/10151(−), similar to primers described in [19].

#### Case Definitions

Diagnoses were considered confirmed if they met any of the following criteria: isolation of virus from the specimen, virus identification by RT-PCR, or 4-fold or greater increase in IgM antibody titers between acute and convalescent samples. Diagnoses were considered presumptive if elevated IgM titers (positive at dilutions greater than or equal to 1∶100) were detected in the acute or in both acute- and convalescent-phase samples without a four-fold rise between samples. Participants with evidence of recent infection by more than one arbovirus are included in each arbovirus category for which such evidence was observed, as the etiologic agent could not be definitively identified. The exception to this was within the *Flavivirus* genus. Since we observed significant IgM cross-reactivity between DENV and YFV antigen (see Results), participants were only considered YFV-positive by serology in the absence of reactivity to DENV antigen in the IgM ELISA. In the absence of laboratory evidence of any of the above pathogens, cases were classified as arbovirus-negative.

### Statistical Analyses

Statistical analyses (Chi-square, Fisher's exact test, and logistic regression) were performed in R version 2.8 (The R Foundation for Statistical Computing, Vienna, Austria)[20]. The significance level was set at α = 0.05.

## Results

A total of 20,880 participants from study sites in Bolivia, Ecuador, Paraguay, and Peru were enrolled in the study between May 2000 and December 2007 (Table 2). A total of 18,201 participants (87.2%; Table 2 were included from Peru, 2,089 (10.0%) from Bolivia, 350 from Ecuador (1.7%), and 240 from Paraguay (1.1%). More than half (10,739; 51.4%) of participants were recruited at 13 health clinics or hospitals in and around Iquitos, Peru. For participants where demographic data was available, 10,919 were male (52.3%) and 9,915 were female (47.5%). The median age of participants was 24 (range 0–92 years), with 89.5% between the ages of 6 and 49. In addition to fever, the most commonly reported symptoms included malaise (96.7%), headache (92.4%), chills (90.2%), myalgia (81.4%), arthralgia (76.2%), and hyporexia (75%). A thorough breakdown of symptomology by etiologic agent will be reported elsewhere (TJK, unpublished results).

**Table 2:** Total febrile participants, by location and year.

Location	2000	2001	2002	2003	2004	2005	2006	2007	Total	% with paired samples
**Bolivia**										
Concepción	-	-	-	-	83	78	113	106	380	75.0
Magdalena	-	-	-	-	70	66	37	-	173	45.6
Santa Cruz	36	101	43	135	93	121	275	476	1280	25.1
Cochabamba	-	-	-	-	-	99	93	64	256	32.0
**Ecuador**										
Guayaquil	-	-	-	2	72	134	82	60	350	86.2
**Paraguay**										
Asunción	-	-	-	-	-	-	18	222	240	48.5
**Peru**										
Piura	1009	404	74	181	65	94	79	78	1984	34.5
Cusco	52	122	133	82	106	95	120	116	826	39.2
La Merced	-	182	140	115	43	69	120	105	774	70.6
P. Maldonado	-	-	-	-	149	318	153	595	1215	42.1
Iquitos	293	897	2113	1040	2308	1458	1433	1197	10739	76.8
Tumbes	147	237	16	107	214	144	153	193	1211	40.0
Yurimaguas	167	124	35	43	227	184	363	309	1452	82.2
**Total**	1704	2067	2554	1705	3430	2860	3039	3521	20880	63.5

Of the 20,880 cases of febrile illness included, paired acute and convalescent-phase samples were collected from 13,259 participants (63.5%), while acute-phase only (without convalescent samples) were available from 7,621 participants (36.5%; Table 2). Most participants for whom data was available reported to a health center within four days following disease onset (15,911/19,632; 81.0%). A subset of participants (n = 9,800; 46.9%) were screened for malaria by thick smear prior to enrollment. Of these, 584 (6.0%) were found to be positive. Patient samples were screened for recent infection by members of the *Flaviviridae*, *Togaviridae*, and *Bunyaviridae* virus families, using IFA and IgM ELISA. Evidence for arbovirus infection was observed in 32.5% of febrile cases overall (Table 3), varying significantly across locations (p<0.001) from 9.3% (Cusco) to 39.8% (Iquitos). Of these, diagnoses were considered confirmed for 4,423 febrile episodes (21.2%), including 2,862 positive by IFA or RT-PCR (13.7%; Table 4) with or without accompanying elevated IgM, as well as 1,561 IgM seroconversions (7.5%) without accompanying IFA or RT-PCR confirmation. No arbovirus co-infections were observed by IFA. An additional 2,370 cases (11.4%) were classified as presumptive arboviral infections. Viral isolation and RT-PCR identification was most successful with participant specimens collected within the first four days following disease onset. Of the 15,911 participants reporting within four days post-disease onset (where data was available), 2,456 (15.4%) were IFA-positive for an arbovirus in the acute sample. For those reporting five days or more post-onset, only 3.8% (143/3,721) were IFA-positive.

**Table 3:** Percentage of febrile participants with evidence of recent infection by one or more arboviruses, by location, 2000–2007.

Location	Pos	Neg	%
**Bolivia**			
Concepción	56	324	14.7
Magdalena	26	147	15.0
Santa Cruz	187	1093	14.6
Cochabamba	78	178	30.5
**Ecuador**			
Guayaquil	109	241	31.1
**Paraguay**			
Asunción	80	160	33.3
**Peru**			
Piura	480	1504	24.2
Cusco	77	749	9.3
La Merced	174	600	22.5
P. Maldonado	373	842	30.7
Iquitos	4276	6463	39.8
Tumbes	394	817	32.5
Yurimaguas	483	969	33.3
**Total**	**6793**	**14087**	**32.5**

Arboviruses include DENV serotypes, YFV, VEEV, MAYV, EEEV, OROV, Group C viruses, and GROV. Full breakdown by virus is provided in Table 5.

**Table 4:** Virus isolates, by location, 2000–2007.

	Flavivirus					Alphavirus		Orthobunyavirus		
**Location**	DENV-1	DENV-2	DENV-3	DENV-4	YFV	VEEV	MAYV	OROV	Group C	GROV
**Bolivia**										
Concepción	0	3	12	0	0	0	2	0	0	0
Magdalena	0	0	1	0	0	0	0	0	0	0
Santa Cruz	0	15	19	0	0	0	7	0	0	0
Cochabamba	0	0	6	0	1	6	6	0	0	1
**Ecuador**										
Guayaquil	3	0	24	1	0	0	0	0	0	0
**Paraguay**										
Asunción	0	0	41	0	0	0	0	0	0	0
**Peru**										
Piura	68	158	22	0	0	0	0	0	0	0
Cusco	0	0	0	0	1	0	3	1	0	0
La Merced	43	0	59	0	0	0	0	0	0	3
P Maldonado	0	39	48	0	1	5	5	1	9	1
Iquitos	66	9	1662	0	1	84	13	16	18	1
Tumbes	45	5	91	3	0	0	0	0	0	0
Yurimaguas	44	1	174	0	0	7	4	0	3	0
**Total**	**269**	**230**	**2159**	**4**	**4**	**102**	**40**	**18**	**30**	**6**

### Malaria co-infections

DENV was the most common malaria co-infection, observed for 11.3% (66/584) of participants reported as malaria-positive, including 17 DENV-3 isolates and one DENV-1 isolate. The rate of DENV infection was significantly lower for malaria-positive participants than for malaria-negative participants (11.3% vs 32.9%; p<0.0001). In contrast, VEEV infection was more common among malaria-positive participants (7.4%) as compared to malaria-negative participants (2.7%; p<0.0001). There were no significant differences between malaria thick smear-positive participants and thick smear-negative participants for the other arboviruses studied (data not shown).

### Flavivirus

DENV serotypes were the predominant arboviruses detected, accounting for 26.0% of febrile episodes analyzed (Table 5), based on virological (2,662 virus isolates or RT-PCR positives, with or without supporting serology) and serological (1,058 IgM seroconversions and 1,700 participants with elevated DENV IgM, without accompanying positive results by virus isolation or RT-PCR) evidence. Considerable YFV cross-reactivity was observed for DENV-positive samples. Based on the 2,662 cases with definitive DENV diagnosis (IFA or RT-PCR confirmation in the acute sample), 847 (32.0%) also had IgM reactive to YFV antigen in the acute or convalescent sample.

**Table 5:** Breakdown of arboviruses associated with febrile illness, by location, 2000–2007.

	DENV			YFV^4^			VEEV			MAYV			OROV			Group C viruses			GROV^5^		
Location	Cnf^1^	Prs^2^	%^3^	Cnf^1^	Prs^2^	%^3^	Cnf^1^	Prs^2^	%^3^	Cnf^1^	Prs^2^	%^3^	Cnf^1^	Prs^2^	%^3^	Cnf^1^	Prs^2^	%^3^	Cnf^1^	Prs^2^	%^3^
**Bolivia**																					
Concepción	20	21	10.8	4	5	2.3	0	1	0.3	3	3		0	0	0.0	0	0	0.0	0	0	0.0
Magdalena	4	7	6.4	4	8	6.9	0	0	0.0	1	2	1.7	0	0	0.0	0	0	0.0	0	0	0.0
Santa Cruz	58	86	11.3	3	24	2.1	1	4	0.4	10	6	1.3	0	2	0.2	0	2	0.2	0	0	0.0
Cochabamba	10	7	6.6	3	17	7.8	11	7	7.0	10	11	8.2	2	7	3.5	2	3	2.0	1	0	0.3
**Ecuador**																					
Guayaquil	55	27	23.4	4	14	5.1	3	5	2.3	1	0	0.3	1	1	0.6	0	3	0.9	0	1	0.3
**Paraguay**																					
Asunción	48	26	30.8	0	5	2.1	0	0	0.0	0	0	0.0	0	0	0.0	0	2	0.8	0	0	0.0
**Peru**																					
Piura	300	176	24.0	0	5	0.3	0	1	0.1	0	0	0.0	0	1	0.1	0	0	0.0	0	0	0.0
Cusco	2	3	0.6	8	50	7.0	0	3	0.4	4	1	0.6	1	3	0.5	0	2	0.2	0	0	0.0
La Merced	125	33	20.4	3	7	1.3	1	0	0.1	0	2	0.2	0	4	0.5	0	1	0.1	5	2	0.9
P. Maldonado	124	86	17.3	22	61	6.8	15	13	2.3	10	10	1.6	7	11	1.5	19	28	3.9	2	1	0.2
Iquitos	2482	965	32.1	65	121	1.7	204	149	3.3	48	59	1.0	65	115	1.7	84	94	1.7	2	4	0.1
Tumbes	189	181	30.6	8	10	1.5	0	6	0.5	0	0	0.0	0	2	0.2	0	0	0.0	1	0	0.1
Yurimaguas	303	83	26.6	19	24	3.0	15	9	1.7	11	5	1.1	4	7	0.8	16	10	1.8	0	1	0.1
**Total**	**3720**	**1701**	**26.0**	**143**	**351**	**2.4**	**250**	**198**	**2.1**	**98**	**99**	**0.9**	**80**	**153**	**1.1**	**121**	**145**	**1.3**	**11**	**9**	**0.1**

1 Confirmed cases (virus isolation, RT-PCR, or IgM seroconversion).

2 Presumptive cases (elevated IgM without 4-fold rise between acute and convalescent).

3 Percent of total febrile cases from this location.

4 Many of the participants with YFV-reactive serum are likely due to recent vaccination or *Flavivirus* cross-reactivity, and thus are not reflective of natural YFV infection (see text).

5 Only limited serologic testing for GROV IgM was performed prior to 2007.

Virus isolates (Table 4) are included with the confirmed diagnoses.

**Figure 2:**
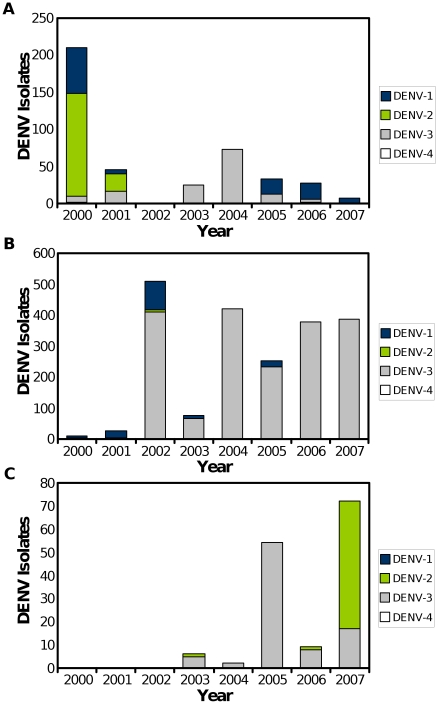
DENV serotypes circulating in western South America, 2000–2007. DENV serotypes circulation was analyzed based on different regions, including (A) Ecuador and northwestern Peru (Tumbes and Piura), (B) northeastern and central Peru (Iquitos, Yurimaguas, and La Merced), and (C) southeastern Peru (Puerto Maldonado) and Bolivia.

DENV-3 was most commonly isolated serotype, accounting for 81.1% (2,159/2,662) of DENV isolates over the course of the study. In our study, DENV-3 was first detected in sites along the northern coast of Peru (Piura and Tumbes) in 2000 (Figure 2) during a large outbreak of dengue fever in the region [21], [22], although DENV-1 and DENV-2 were the most commonly isolated serotypes during this outbreak. DENV-3 quickly became the dominant serotype in the northeastern rainforest (Iquitos and Yurimaguas), with limited DENV-1 co-circulation in the region in subsequent years (Figure 2). Between 2002 and 2006 little DENV-2 transmission was observed until DENV-2 emerged in the study sites in Bolivia and southern Peru (Puerto Maldonado) in 2007 (Figure 2). DENV-4 was rarely detected during the study period, with only four isolates from study participants. However, more recently this situation has changed dramatically with the 2008 emergence of DENV-4 in northern Peru [23].

Overall, DENV infection was more common among female participants than male participants (28.1% vs 25.5%; p<0.0001), with a statistically significant bias towards older participants (28.0% of participants 30 or older were DENV-positive as compared with 25.9% of participants younger than 30; p = 0.001). However, the epidemiology of DENV infection varied by study site, particularly within Peru. The prevalence of DENV infection was higher among older participants in Puerto Maldonado (p = 0.01), La Merced (p<0.0001), Piura (p<0.0001), and Tumbes (p = 0.015), while no elevated DENV prevalence was observed for older participants in Yurimaguas and Iquitos. In addition, DENV infection was more common among female participants in Puerto Maldonado (p<0.0001), La Merced (p<0.0001), and Tumbes (p = 0.028), but no statistically significant difference was observed in Iquitos, Yurimaguas, or Piura.

Other than DENV, the only flavivirus isolated during the course of the study was YFV, which was isolated from four participants (Table 4). In addition to the four isolates, serological evidence of recent YFV infection (without evidence of DENV infection) was detected in an additional 494 participants, including 143 who seroconverted between acute and convalescent samples. Overall, data on prior vaccination was available for 17,816 participants, 10,667 (59.9%) of whom reported having received YF vaccination. YF vaccine coverage varied widely by study site, ranging from less than 10% in non-endemic sites along the northern coast of Peru (Piura and Tumbes) to 59% in Iquitos, 70% in Yurimaguas, and 77% or greater in Cochabamba (Villa Tunari and Eterazama), Concepción, Junin, Magdalena, and Puerto Maldonado. Study participants with evidence of recent YFV infection based on IgM were significantly more likely than the rest of the overall study population to have reported receiving YF vaccination within the previous 6 months (30.0% vs. 6.7%; p<0.0001).

### Alphavirus

Recent alphavirus infection was detected for 3.1% (n = 645) of febrile patients (Table 5), including 102 VEEV isolates and 40 MAYV isolates. RNA from a subset of VEEV isolates was extracted, reverse transcribed, amplified, and sequenced. All sequenced isolates were determined to belong to enzootic subtypes of the VEE complex, predominately ID Panama/Peru or Peru/Bolivia genotypes [12], [14], [24] although there was one ID Colombia/Venezuela genotype and one IIID subtype virus identified, both in Iquitos [12]. The majority of VEEV isolates and seroconversions (234/250; 93.6%) were from patients in Iquitos, Puerto Maldonado, and Yurimaguas, Peru. In contrast, MAYV isolates were more prevalent in Bolivia and southeastern Peru (Table 4). Of all MAYV isolations, 57.5% (23/40) were from this region, despite representing only 19.8% of all participants in the study.

EEEV was not isolated from any participant samples during the course of the study. A subset of participant samples were screened for EEEV-reactive IgM (n = 3,014), with serological evidence for EEEV infection in 22 cases (0.7%), including two seroconversions. Unlike the flaviviruses, little serologic cross-reaction (or, alternatively, concurrent infection) was observed among alphaviruses. For the 72 participants with the most well-defined VEEV infections (IFA-positive, plus a convalescent sample available for testing), 7 (9.7%) had IgM reactive to MAYV antigen in either the acute or convalescent sample. For the 24 cases where MAYV was isolated and a convalescent sample was available for testing, no cross-reactivity was observed in the VEEV IgM ELISA.

### Orthobunyavirus

Arboviruses belonging to the *Orthobunyavirus* genus of the *Bunyaviridae* family accounted for approximately 2.5% of all febrile cases (Table 5). In total there were 54 orthobunyavirus isolates, including 30 Group C viruses, 18 OROV isolates, and six GROV (Table 4). The Group C virus isolates were not definitively identified; however, based on serological techniques (ELISA and PRNT), ten were antigenically related to CARV and six were antigenically related to MURV, while 14 could not be antigenically distinguished. Nearly all Simbu Group (OROV) and Group C virus isolates were collected from patients reporting to clinics in Iquitos, Madre de Dios, and Yurimaguas, Peru, while three out of six GROV isolates were obtained from patients in La Merced, Peru, in January and February of 2007 (Table 4). As with the alphaviruses, little serologic cross-reaction was observed within the *Orthobunyavirus* genus. For the 24 participants IFA-positive for a Group C virus and a convalescent sample available, only 2 had IgM reactive to OROV antigen in either the acute or convalescent sample (8.3%); for the 12 OROV IFA-positive participants with a paired convalescent sample, no reactivity with CARV or MURV antigen was observed.

### Incidence rates and epidemiology of arbovirus infection in Iquitos, Peru

The Iquitos health centers included in this study cover a geographically stratified area of the city and in 2007 represented nearly 20% (10 of 55) of civilian public health centers in the greater urban health network. Based on the populations assigned to each health center by the local ministry of health (Dirección Regional de Salud -Loreto), in 2007 clinics included in this study were designated to serve approximately 43% of the population of the Iquitos region. Using the population numbers assigned to each health center by DIRESA-Loreto, we estimated incidence rates for the most common arboviral infections in Iquitos beginning with the first full year of the study (Table 6). Over the course of the study, there were 855.9 acute undifferentiated febrile episodes per 100,000 people per year, peaking during periods of highest dengue activity (2002 and 2004; Table 6). DENV incidence rates varied greatly, peaking in 2002 with 554.0/100,000 following the introduction of DENV-3 and averaging 274.7/100,000 over the 7-year period. The average symptomatic incidence rates for other predominant arboviruses were 28.1/100,000 for VEEV, 8.5/100,000 for MAYV, 14.3/100,000 for OROV, and 14.2/100,000 for Group C viruses. Peak transmission rates were observed for these four viruses between 2004 and 2006, including a previously-described outbreak of VEEV in 2006 [24].

**Table 6:** Yearly incidence rates of arboviral diseases in Iquitos, Peru, per 100,000 residents.

	2001	2002	2003	2004	2005	2006	2007	Average
All Febrile Cases	519.5	1213.2	591.9	1277.1	799.9	779.6	645.7	**855.9**
DENV	10.4	554.0	83.1	498.0	242.5	294.3	231.4	**274.7**
VEEV	16.2	16.1	14.2	25.5	30.7	58.2	24.8	**28.1**
MAYV	2.3	6.9	5.1	10.0	9.3	20.1	3.8	**8.5**
OROV	0.0	4.0	3.4	17.7	27.4	41.9	3.8	**14.3**
Group C viruses	1.2	2.9	5.1	30.4	33.5	12.0	11.9	**14.2**

The first year (2000) is excluded as the study did not start until mid-way through the year.

It should be noted that the incidence rates above only reflect the participants enrolled in the study. Starting in 2006, demographic data was collected for those who reported to Iquitos health centers and fulfilled the inclusion criteria (acute undifferentiated febrile illness of fewer than 7 days in duration) but declined participation in the febrile surveillance study. In 2006 and 2007, 3,385 and 3,283 febrile patients, respectively, fitting the case definition were examined by study personnel, with 43.3% (n = 1,433) and 36.5% (n = 1,197) of patients agreeing to provide venous blood samples for the surveillance program. During these two years, 94.3% of febrile patients were first screened for malaria by thick smear, with 32.4% of those screened classified as positive. Malaria-negative patients were significantly more likely to accept participation in the surveillance study (51.3%; 2,185/4,256) than malaria-positive patients (9.5%; 193/2,036; p<0.001). Children were significantly less likely to participate than adults (25.3% of eligible children vs. 44.0% of eligible adults chose to participate; p<0.001).

To begin to describe the epidemiology associated with these arboviruses in the Iquitos, demographic characteristics of participants with recent infection by the most common pathogens – DENV serotypes, VEEV, MAYV, OROV, and Group C viruses – were compared with the rest of the participating febrile population in Iquitos. YFV infection, as determined by positive IgM ELISA, was significantly associated with self-reported recent YF vaccination (OR 2.30, 95% CI 1.44—3.57); no similar association was observed for other arboviruses. Thus no further analyses were conducted for YFV IgM-positive participants. Overall, male participants were more common than female (51.4% vs. 48.6%), consistent with the population of Loreto Department as a whole (51.2% male; p = 0.77)[25]. The median age of study participants was 23 (average 26.1), with the highest percentage of participants between the ages of 15 and 29. Both MAYV (p = 0.003) and VEEV (p = 0.009) infection were significantly more common among males, and this effect was only observed among the older age groups (15 years or older), suggesting an occupational exposure. A similar trend for higher prevalence of *Alphavirus* infection among males was observed in Yurimaguas and Puerto Maldonado, although these analyses were limited by small sample size. Group C virus infection was more common in males in these three sites, although the differences were only statistically significant in Puerto Maldonado (p<0.01). In Iquitos no significant differences were observed between sexes for DENV or OROV (Table 7). DENV infection was significantly more common among participants younger than 15 in Iquitos (p = 0.005); however, this effect was only observed during the earlier years of the study (2002 and 2003 in particular). OROV infection in Iquitos was significantly more common among age groups 15 or older (p = 0.007). For the 30–44 year old age group, MAYV infection was significantly more common than for participants younger than 15 (Table 7).

**Table 7:** Association between arbovirus infection and demographic variables in Iquitos, Peru, 2000–2007.

Variable	Category	Total	DENV	VEEV	MAYV	OROV	Group C viruses
		No. (%)	No. (% pos)	No. (% pos)	No. (% pos)	No. (% pos)	No. (% pos)
Sex	Male	5519 (51.4)	1764 (32.2)	206 (3.7)*	71 (1.3)*	94 (1.7)	103 (1.9)
	Female	5220 (48.6)	1683 (32.0)	147 (2.8)	36 (0.7)	86 (1.6)	75 (1.4)
Age group	<15	2175 (20.2)	751 (34.5)*	61 (2.8)	15 (0.7)	21 (1.0)*	27 (1.2)
(yrs)	15–29	4959 (46.2)	1557 (31.4)	156 (3.1)	45 (0.9)	80 (1.6)	81 (1.6)
	30–44	2311 (21.5)	748 (32.4)	95 (4.1)	28 (1.2)	54 (2.3)	47 (2.0)
	45+	1290 (12.0)	389 (30.2)	41 (3.2)	19 (1.5)	25 (1.9)	23 (1.8)
Clinic	Urban	8552 (79.6)	3080 (36.0)**	271 (3.2)*	70 (0.8)**	139 (1.6)	128 (1.5)**
	Military	1049 (9.8)	291 (27.7)	29 (2.8)	16 (1.5)	23 (2.2)	15 (1.4)
	Rural	1138 (10.6)	76 (6.7)	53 (4.7)	21 (1.8)	18 (1.6)	35 (3.1)

*p<0.05;

**p<0.001.

For the Total column, numbers in parentheses represent the distribution of each variable within the category. For each individual arbovirus the numbers in parentheses represent the percent of participants within each variable category that were positive for the selected arboviruses.

The health centers in the Iquitos area included in this study were predominantly public clinics and hospitals located within the urban area of the city (n = 8), although the study was also conducted in three military clinics located within the urban area and two public clinics located in rural zones between approximately five and ten kilometers outside the city limits. The majority of participants in the Iquitos area were recruited in the urban clinics (79.6%), while 10.6% and 9.8% were recruited at the two rural clinics and three military clinics, respectively. Using the different categories of clinics as a proxy for potential differences in arbovirus exposure, we compared the relative prevalence of arboviruses among those reporting to the urban, rural, or military clinics. DENV infection was far more common in participants reporting to the urban clinics than rural clinics, whereas VEEV (p = 0.018), MAYV (p<0.001), and Group C viruses (p<0.001) were more common among those reporting to the rural clinics. For OROV infection there was no statistically significant differences among the types of health centers (Table 7).

**Figure 3:**
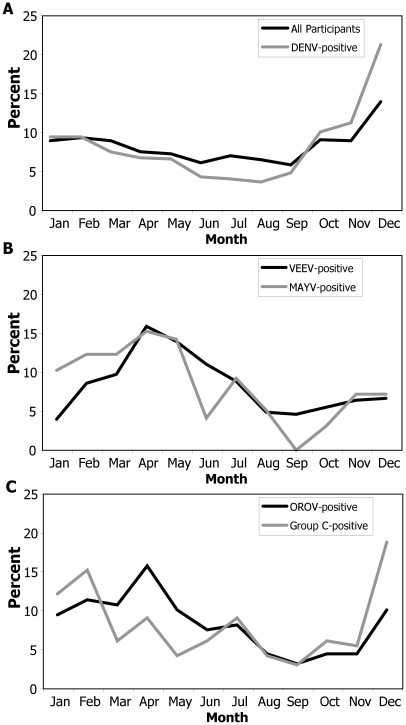
Monthly distribution of participants with arbovirus infection in Iquitos, Peru, 2000–2007. Data are represented as the percent of participants with date of disease onset in each month. The monthly distribution of all participants is shown in (A), while participants with recent infection by a DENV, an alphavirus, or an orthobunyavirus are shown in (A), (B), and (C), respectively.

Participants were recruited year-round, with a peak in December that was largely due to DENV transmission (Figure 3A). Transmission of the arboviruses peaked during different months of the year. Over the course of the study, DENV transmission was most common between October and December with lowest levels between June and August (Figure 3A). Alphavirus transmission was highest between February and July (Figure 3B), with reduced transmission during the second half of the year, while the highest percentage of Group C virus cases was observed between December and February (Figure 3C).

## Discussion

Tropical areas, with year-round hot and humid conditions, are particularly well-suited for maintaining arboviruses with both current public health importance as well as the potential to emerge as significant human pathogens [4]. Therefore in this study we focused on arbovirus transmission in tropical regions of four countries in South America. Our data demonstrate that arboviruses are a common cause of human febrile illness in these sites in South America, accounting for greater than 30% of the febrile episodes analyzed. Importantly, arbovirus-associated disease was not restricted to DENV in most of the locations studied. The other arboviruses identified, including VEEV, MAYV, and OROV, in total were associated with approximately 8% of febrile episodes. Our study has provided source material for various phylogenetic analyses [12], [14], [19], [23], [26] and will provide important baseline data for monitoring changes in arbovirus ecology, epidemiology, and genetics.

There were several significant limitations to our study. First, with the exception of Peru, the number of study sites in the other countries was quite limited. Even in Peru, it is unclear whether these results are indicative of arbovirus circulation in other regions of the country. Another shortcoming was the focus on arboviruses. While clearly these are important pathogens in the tropical rainforest regions, along the desert coast (Piura) and in the highlands (Cusco), other types of pathogens will need to be given greater consideration. Another limitation of our study design is the passive surveillance strategy employed. Clinic-based surveillance is likely to significantly underestimate true arbovirus circulation, as those with milder disease manifestations are less likely to visit a health center. In studies of DENV transmission in Iquitos we have observed that incidence rates calculated from community-based active surveillance are several times higher than those calculated based on passive surveillance (TJK, ACM, and BMF, unpublished results). Accordingly the incidence rates presented here should be interpreted carefully and considered a conservative estimate of the true number of febrile episodes caused by each virus. Another shortcoming of clinic-based surveillance is the difficulty of extrapolating the data to the entire population. As we show here in Iquitos, those who present to the health centers and those that are willing to participate in these studies are often not fully representative of the population at-large, which may lead to biases in age-dependent incidence rates. In addition, with the exception of Iquitos, we did not collect sufficient data from non-participants to fully contextualize these results. Our data suggest that malaria may contribute to approximately 30% of acute febrile illnesses in Iquitos, a figure that would not be apparent based solely on those who enrolled in the study. One advantage of clinic-based passive surveillance is expanded geographic coverage and more limited costs relative to other surveillance strategies, which is critically important when studying the relatively obscure arboviruses described here. Only through the large number of participants presented here were we able to detect sufficient cases of VEEV, MAYV, and OROV for further epidemiological analysis.

Over the course of the study, DENV serotypes were by far the most common arboviruses associated with febrile disease, accounting for 26% of febrile participants. DENV serotypes have emerged dramatically in Latin America over the past decades, to the point that nearly a million cases of dengue fever are reported every year in Latin America, along with thousands of cases of more severe disease that may lead to hemorrhagic manifestations and death [27], [28]. Here we demonstrate that DENV-3 (previously identified as subtype III [26]) was the predominant serotype in the region between 2001–2007, although we also observed significant DENV-1 and DENV-2 transmission in certain regions. Not surprisingly DENV circulation was found to be more region-dependent than country-dependent. Specifically, Tumbes and Piura along the coast of northern Peru share common trends with Guayaquil in Ecuador (Figure 2A), while DENV circulation in Puerto Maldonado in southern Peru is more closely tied to trends observed in Bolivia (Figure 2C). More recently we observed that a genetically conserved strain of DENV-4 was identified in Ecuador (2006), then coastal Peru (2007), before spreading to the tropical rainforests of northeastern Peru (Iquitos and Yurimaguas; 2008) [23]. As multiple serotypes have been circulating in the region severe disease resulting from heterologous secondary infection is increasingly likely to occur [27], [29]. In this study we did not distinguish between primary and secondary infection, and thus further analysis will be needed to identify the genotypes [30] and prior DENV immune status associated with more severe disease outcomes in the region. Regardless, the data described here will provide a springboard for future studies of regional DENV maintenance and dispersion patterns [31] as well as analysis of genetic adaptation and selective pressures.

Other than DENV, the only other flavivirus isolated was YFV. One well-documented hindrance to study flavivirus is the cross-reaction observed among even disparate species [32], [33]. Similarly here we observed significant cross-reaction between DENV and YFV antigen in serum from patients with defined DENV infection, thus there is a possibility that some of the cases have been misclassified. For YFV, we only considered those instances where there was no DENV IgM detected. Furthermore, there was a strong correlation between participants reporting recent YF vaccination and having YFV-reactive IgM, suggesting that these results were not due to cross-reactivity with other flaviviruses circulating in South America. Low grade fever and headache are not uncommon outcomes within the two weeks following YF vaccination [34], [35], so it is possible that these cases are due to the vaccination. It should be noted, however, that flavivirus IgM can be long-lived [32], and thus many of these febrile episodes classified as “YFV infection” may not represent the true etiologic agent. In addition, in our study we only rarely observed severe disease associated with YFV-reactive IgM, suggesting that these cases largely do not reflect natural infection and thus should be interpreted with caution.

In addition to DENV and YFV, there are other flaviviruses circulating in the region that need to be considered, including WNV, Rocio virus (ROCV), Ilheus virus (ILHV), and St. Louis encephalitis virus (SLEV). These flaviviruses have been isolated either from mosquitoes [36], birds [37], or mammals [38], including humans [39], [40], in parts of South America. More closely related to our study areas, ILHV has been isolated from a febrile patient in Ecuador [41], and ILHV and SLEV have been isolated from mosquitoes in Iquitos [36], clearly demonstrating that these viruses are circulating near human populations in the region. None of these viruses were isolated from participants in our study, suggesting that human infection is uncommon. However, in a preliminary survey of a subset of our participants we have identified cases where ROCV, ILHV, SLEV, or WNV IgM was detected, with no reactivity with DENV or YFV antigen (data not shown), with confirmation by virus neutralization assay, considered the most specific tool for flavivirus serology [42]. Overall, the cross-reactivity reported here and elsewhere [32] and the longevity of flavivirus IgM underscore the complications of flavivirus serodiagnosis, which represents a great hindrance for epidemiological surveillance.

The most common *Alphavirus* species identified were VEEV and MAYV. Scant evidence for human infection with EEEV was identified, consistent with previous reports [43], despite evidence of EEEV circulation in mosquitoes near Iquitos [36], [44], for example. In light of the recent emergence of another alphavirus, CHIKV, in the Indian Ocean region, VEEV and MAYV represent interesting cases to consider with regards to potential for urban emergence. In laboratory studies *Aedes* spp., the primary vectors for DENV, have been shown to be a competent vector for VEEV [45] and MAYV [46]. Even without adapting to human-*Aedes*-human cycles, epizootic VEEV subtypes have been associated with large outbreaks of human disease across South America [6]. As recently as 1995 a VEEV outbreak was responsible for nearly 100,000 febrile cases in Venezuela and Colombia [47], [48]. While the VEEV strains isolated in our study all belong to enzootic genotypes of the virus complex [12], [14], [24], genetic studies have demonstrated that enzootic and epizootic subtypes are closely related. A modest number of amino acid changes can alter the viral phenotype dramatically, converting an enzootic strain to an epizootic strain [49]–[52]. Similarly, amino acid variants in the CHIKV E1 protein have been associated with increased epidemic potential [53]–[55]. Several other factors further suggest that potential for neotropical alphavirus emergence is high. In the Iquitos area, while we found that VEEV was more commonly associated with rural clinics (Table 7), many of the participants with confirmed VEEV infection lived within the city and did not report leaving the urban area during the month prior to the febrile illness [24](data not shown). This data is corroborated by a previous study of healthy participants, in which we found that nearly 25% of the urban population carries VEEV-neutralizing antibodies [24]. In addition, based on data collected through this program the geographic range of MAYV and VEEV is wider than had been previously demonstrated, extending to southern Peru and Bolivia [14], [19]. Taken together these factors suggest that the potential establishment of the neotropical alphaviruses as urban pathogens should be closely monitored.

In addition to the flaviviruses and alphaviruses, orthobunyaviruses were significant sources of febrile illness in the study, accounting for 2.5% of febrile episodes analyzed. While all orthobunyavirus isolates came from patients in Peruvian rainforest sites during the course of this study, we did find serological evidence for OROV and Group C viruses in Ecuador and Bolivia. More recently (2008) we have definitively identified Group C viruses in Bolivia, isolated from two participants in the Cochabamba region (data not shown). Like VEEV and MAYV, OROV is an interesting case study with regards to potential for broader emergence. OROV has been associated with numerous outbreaks in Brazil, infecting approximately 500,000 people in South America over the past 45 years [56]–[58]. Two distinct transmission cycles have been proposed, a poorly-defined sylvatic cycle and an urban cycle where OROV is transmitted among humans by the biting midge *Culicoides paraensis* [58], [59]. In Iquitos, we found that unlike the Group C viruses, VEEV, and MAYV, evidence of recent OROV infection (based on both IgM and virus isolation data) showed no significant bias towards rural clinics, suggesting similar transmission levels between urban and rural sites, consistent with results from an earlier survey of healthy participants in the region [10]. This pattern may reflect a peri-urban transmission cycle, as the majority of the OROV isolates were detected in both the rural sites and an urban site located towards the periphery of the city in 2005 and 2006 during a period of markedly increased transmission (Table 6). OROV isolates from previous Iquitos studies (prior to 1998) were found to belong to lineage II, similar to strains associated with Brazilian OROV outbreaks [56], [60]. Future sequence analysis of the more recent isolates described in this current study from Iquitos, Yurimaguas, and Puerto Maldonado, will provide a more complete description of OROV geographic and temporal genetic variability.

Considering the association of arboviral pathogens with human disease and the potential for wider-scale emergence, disease surveillance is an integral component of public health planning, disease control, and analysis of potential intervention strategies. Unfortunately, for the arboviruses described here syndromic surveillance is complicated by the lack of pathogen-specific signs and symptoms [5], particularly early in disease progression. As with other reports [7], [9], our study underscores the need for laboratory-based support of febrile surveillance studies. Even within our study other pathogens clearly need to be considered, as the majority of febrile episodes in this study were not associated with an arboviral etiology. In Iquitos past studies have linked both *Leptospira* spp. and *Rickettsia* spp. with a significant percentage of febrile illnesses [61]–[63]. To-date, solid data are lacking for the other study sites included in this study, although our preliminary results suggest that *Leptospira* spp. and *Rickettsia* spp. are common human pathogens in these locations as well (TJK, unpublished results). Admittedly our studies provide little in the way of guidance for patient care but do point toward the need for the development of pharmaceutical therapies for the treatment of a variety of viral infections. In addition the development of rapid and inexpensive diagnostic tools should be given high research priority, particularly to distinguish arbovirus infection from other pathogens where effective and inexpensive pharmaceutical treatment is already available, such as for *Rickettsia* spp. and *Leptospira* spp.

## Acknowledgments

### NMRCD Febrile Surveillance Working Group

#### Bolivia

Roberto Agudo (SEDES-Cochabamba), Renato Amonzabel (Hospital de Jorochito), Omar Camargo (SEDES Trinidad), Simar Del Villar (Hospital Municipal César Banzer), Simon Delgado (Centro de Salud, Eterazama), Roxana Loayza (CENETROP, Santa Cruz), Agripina Mamani (Red de Servicios de Villa Tunari), Miguel Montero (Hospital Municipal César Banzer), David Paz (Hospital El Torno, Santa Cruz), Yelin Roca (CENETROP, Santa Cruz), Fernando Terrazas (Hospital de Villa Tunari), Jaime Vargas Yapura (Hospital San Francisco de Asís, Villa Tunari)

#### Paraguay

Alma Barboza (ONG “Rayos de Sol”, Asunción), Liliana Giménez de Sosa (ONG “Rayos de Sol”, Asunción), Maria Eugenia León (ONG “Rayos de Sol”, Asunción), Marta Terol (ONG “Rayos de Sol”, Asunción)

#### Ecuador

Araceli Alava (INHMT “Leopoldo Izquieta Pérez”, Guayaquil), Franklin Delgado (Hospital Naval, Guayaquil)

#### Peru

Javier Acha (Clínica Naval, Iquitos), Jorge Aldazabal (Dirección Regional de Salud de Madre de Dios, Puerto Maldonado), Elizabeth Anaya (Instituto Nacional de Salud, Lima), Irene Anaya (Hospital Santa Rosa, Puerto Maldonado), Alberto Ancasi (Hospital Militar Regional Santa Rosa, Iquitos), Jackeline Aspajo (NMRCD/Hospital 2 de Mayo, Lima), Karla Block (NMRCD, Iquitos), César Cabezas (Instituto Nacional de Salud, Lima), Rebeca Carrión (NMRCD, Iquitos), Omayra Chincha (NMRCD/Hospital General La Merced, Chanchamayo), Mariangela Duffoó (NMRCD/Hospital General La Merced, Chanchamayo), Eduardo Falconi (Instituto Nacional de Salud, Lima), Connie Fernández (Hospital Santa Gema, Yurimaguas), Jorge Gómez (Dirección General de Epidemiología, Lima), Juliany Granda (Centro de Salud de Chiclayito, Piura), Eric Hall (NMRCD, Lima), Carlos Holguín (Laboratorio Referencial, Piura), Andrés Lescano (NMRCD, Lima), Percy Minaya (Dirección General de Epidemiología, Lima), Silvia Montano (NMRCD, Lima), Dora Ines Nakanishi (Hospital La Merced, La Merced), César G. Náquira (Instituto Nacional de Salud, Lima), Víctor Ocaña (Centro de Salud – Chiclayito, Piura), Paul Pachas (Dirección General de Epidemiología, Lima), Fernando Quintana (Ministerio de Salud, Tumbes), Tatiana Saldarriaga (NMRCD/Laboratorio Referencial, Tumbes), Luis Sánchez H. (Universidad Nacional Mayor de San Marcos, Lima), Moisés Sihuincha (DISA Loreto, Iquitos), Giannina Suaña (Hospital La Merced, La Merced), Luis Suárez (Dirección General de Epidemiología, Lima), Robert Tesh, (University of Texas Medical Branch, Galveston, Texas), Carlos Vidal (DISA-Loreto, Iquitos), Stalin Vilcarromero (NMRCD/Hospital Santa Gema, Yurimaguas), Miguel Villanueva (NMRCD/Hospital Regional, Cusco), Hernán Zamalloa (NMRCD/Hospital Santa Rosa, Puerto Maldonado)

#### NMRCD Laboratory personnel

Alfredo Huaman, Cecilia Rivera, Roger Castillo, Christian Albujar, Cristhopher Cruz, Vidal Felices, Roxana Caceda, Juan Sulca, Zonia Rios, Angelica Espinoza, Diana Juarez, Elizabeth Castillo, Alicia Rosas, Pedro Palermo, Wieslawa Alava, Guadalupe Flores, Leslye Angulo

#### NMRCD field personnel

Rubi Rubio, Lucy Navarro, Marcelina Flores, Johnni Mozombite, Iris Reategui, Zenith Pezo, Juan Flores, Magali Ochoa, Zoila Reategui, Geraldine Ocmin, Leny Curico, Regina Fernandez, Nora Marin

Disclaimer: The views expressed in this article are those of the author and do not necessarily reflect the official policy or position of the Department of the Navy, Department of Defense, nor the U.S. Government.

Some authors of this manuscript are military service members or employees of the U.S. Government. This work was prepared as part of their official duties. Title 17 U.S.C. § 105 provides that ‘Copyright protection under this title is not available for any work of the United States Government’. Title 17 U.S.C. § 101 defines a U.S. Government work as a work prepared by a military service members or employees of the U.S. Government as part of those person's official duties.
